# The alarmin–ILC2 axis as a candidate mechanism for persistent olfactory dysfunction in allergic rhinitis

**DOI:** 10.3389/fimmu.2026.1867859

**Published:** 2026-07-08

**Authors:** Jin-xiang Zhu, Hao-ran Luo, Dan Li, Biao-qing Lu, Wei-juan Zhang, Guan-jiang Huang, Yan Ruan

**Affiliations:** 1 Department of Otorhinolaryngology Head and Neck Surgery, Zhongshan Hospital of Traditional Chinese Medicine, Affiliated to Guangzhou University of Chinese Medicine, Zhongshan, Guangdong, China; 2 The Tenth Clinical Medical College of Guangzhou University of Chinese Medicine, Zhongshan, Guangdong, China; 3 The First Clinical Medical College of Guangzhou University of Chinese Medicine, Guangzhou, Guangdong, China; 4 Department of Otorhinolaryngology, Shenzhen Traditional Chinese Medicine, Shenzhen, Guangdong, China; 5 Department of Otorhinolaryngology, Shaoguan Traditional Chinese Medicine, Shaoguan, Guangdong, China; 6 Department of Otolaryngology Head and Neck Surgery, The First Affiliated Hospital of Guangzhou University of Chinese Medicine, Guangzhou, Guangdong, China; 7 Lingnan Institute of Otolaryngology, Guangdong Clinical Research Academy of Chinese Medicine, Guangzhou, Guangdong, China

**Keywords:** alarmin, allergic rhinitis, horizontal basal cells, IL-33, ILC2, olfactory cleft, olfactory dysfunction, sensorineural

## Abstract

**Background:**

Allergic rhinitis (AR) is an underrecognized contributor to olfactory dysfunction (OD). Although smell loss in AR is commonly attributed to conductive obstruction, some patients exhibit persistent or disproportionate hyposmia despite improvement in nasal congestion, suggesting that additional sensorineural mechanisms may exist.

**Hypothesis:**

We hypothesize that, in a subset of patients with persistent, moderate-to-severe, type 2-high AR-associated OD without macroscopic olfactory cleft obstruction, allergen-induced epithelial stress in the olfactory cleft may activate an alarmin–ILC2/type 2 neuroimmune pathway. IL-33 and thymic stromal lymphopoietin (TSLP) released from epithelial or sustentacular cell populations, together with IL-25 and lipid mediator signals from tuft-like microvillar cells, may engage group 2 innate lymphoid cells (ILC2s) as early amplifiers within a broader type 2 inflammatory network. This response is proposed to contribute to three candidate injury modules: IL-13/STAT6-associated horizontal basal cell fate bias, IL-5/eosinophil-associated olfactory sensory neuron injury, and IL-4/IL-13-associated sustentacular cell dysfunction.

**Rationale and evidence gap:**

Current support is indirect and derives from murine olfactory inflammation models, ex vivo olfactory preparations, human non-olfactory nasal mucosal studies, and chronic rhinosinusitis with nasal polyps (CRSwNP) studies used as mechanistic analogies. None of the proposed mechanisms has been directly validated in human AR olfactory cleft tissue, which together represent the central evidence gap.

**Implications:**

The model generates falsifiable predictions regarding olfactory cleft alarmins, type 2 cytokines, ILC2-like immune niches, basal cell transcriptional state, and biomarker-linked responses in future proof-of-mechanism studies. It is presented as a hypothesis to guide future non-invasive biomarker studies, ethically feasible tissue analyses, organoid experiments, and biomarker-enriched proof-of-mechanism trials, not as a validated disease mechanism or current treatment recommendation.

## 1 Introduction

### 1.1 AR-associated olfactory dysfunction as an underrecognized clinical problem

Allergic rhinitis (AR) is a highly prevalent inflammatory airway disease, with reported prevalence ranging from approximately 10% - 40% depending on region, age group, and diagnostic criteria. Although sneezing, rhinorrhea, nasal itching and congestion are its most recognized manifestations, AR also affects sleep, cognition, work productivity and quality of life (1). Olfactory dysfunction (OD) is increasingly recognized as an important but underassessed clinical problem in AR. When objective psychophysical tools such as the Sniffin’ Sticks threshold–discrimination–identification (TDI) score or the University of Pennsylvania Smell Identification Test (UPSIT) are used, OD has been reported in approximately 28%-43% of patients with AR (2, 3). Smell loss is clinically meaningful because it impairs detection of environmental hazards, affects nutrition and food enjoyment, and is associated with reduced psychological well-being and quality of life (2, 4).

Despite this burden, olfactory function is not routinely quantified during standard AR assessment, and evidence-based management strategies specifically targeting AR-associated OD remain limited. This gap between clinical impact and mechanistic understanding motivates the hypothesis developed in the present article.

### 1.2 Why conductive obstruction may not fully explain persistent AR-associated OD

The conventional explanation for AR-associated OD is conductive: mucosal edema, turbinate swelling, and altered nasal airflow may reduce odorant access to the olfactory cleft and thereby impair stimulation of olfactory sensory neurons (OSNs) (5). This mechanism is clearly relevant, particularly in mild or intermittent AR with fluctuating congestion. However, if conductive obstruction were the only mechanism, improvement in nasal airflow would be expected to restore olfactory function proportionally in most patients.

Several clinical observations suggest that this relationship is incomplete in at least a subset of patients. *Stuck* and *Hummel* reported variable and often incomplete olfactory improvement after intranasal corticosteroid (INCS) treatment in AR (5). *Guilemany* et al. found that smell impairment in persistent AR was related not only to congestion but also to local inflammatory features (6). Recent reviews also indicate that olfactory impairment may correlate only weakly with objective or radiological indices of nasal obstruction in mixed inflammatory cohorts, although these findings should be interpreted cautiously because AR and chronic rhinosinusitis (CRS) populations are often combined (2, 3). *Klimek* and *Eggers* further showed that olfactory dysfunction in AR was associated with nasal eosinophilic inflammation, supporting the possibility of an inflammatory contribution beyond airflow limitation (7).

Taken together, these observations do not refute the conductive model, but they suggest that conductive obstruction alone may not fully explain persistent or disproportionate OD in some patients with AR. A complementary sensorineural hypothesis may therefore be useful. The comparative clinical and mechanistic profile of AR relative to CRSwNP and post-viral OD is summarized in **Table 1**.

**Table 1:** Clinical and mechanistic contrasts among inflammatory olfactory disorders, with evidence level indicated for AR-OD.

Parameter	Allergic rhinitis	CRSwNP	Post-viral OD
Primary OD mechanism	Conductive mechanism established; putative sensorineural component in persistent type 2-high AR-OD	Conductive (dominant) and sensorineural	Sensorineural
Dominant inflammatory endotype	Heterogeneous; type 2-high/eosinophilic endotype most relevant to this hypothesis	Type 2 eosinophilic (Western)	Viral cytopathic/neuroimmune
Macroscopic polypoid obstruction	Absent	Present	Absent
OE structural remodeling	Hypothesized microscopic remodeling; not demonstrated in human AR olfactory cleft	Severe (neuron depletion, polyp formation)	Variable (sustentacular cell loss)
Reported OD prevalence	28% - 43%	60% - 83%	Up to 60% (acute)
Correlation of OD with congestion score	Variable; weak in some mixed inflammatory cohorts; AR-specific data limited	Moderate	Not applicable
Response to INCS (TDI improvement)	Variable; often improves conductive symptoms but may not fully restore smell in some patients	Moderate	Poor
Biologic therapy OD evidence level	No direct AR-OD trial; indirect hypotheses derived from CRSwNP and type 2 airway data	Established (dupilumab, mepolizumab, tezepelumab)	Not applicable
Key alarmin biomarker in secretions	Predicted: IL-33, TSLP, IL-25; olfactory cleft-specific AR data absent	IL-33, TSLP, IL-25	Not characterized
Olfactory neurogenesis status	Predicted impairment of neurogenesis; direct human AR olfactory cleft evidence absent	Severely impaired (HBC immune switch)	Impaired (GBC depletion)

This table summarizes broad differences among inflammatory causes of OD. For AR, the proposed sensorineural mechanism remains hypothetical and applies primarily to persistent, type 2-high AR-OD without nasal polyps or macroscopic olfactory cleft obstruction. CRSwNP biologic data are not direct evidence for AR-OD because polyp reduction and airflow improvement confound olfactory outcomes. AR, allergic rhinitis; CRSwNP, chronic rhinosinusitis with nasal polyps; HBC, horizontal basal cell; ILC2, group 2 innate lymphoid cell; INCS, intranasal corticosteroid; OD, olfactory dysfunction; TDI, threshold–discrimination–identification composite score; Th2, type 2 helper T cell; TSLP, thymic stromal lymphopoietin; UPSIT, University of Pennsylvania Smell Identification Test.

### 1.3 Scope and aim of this hypothesis article

This article proposes a candidate mechanistic framework for the sensorineural component of persistent AR-associated OD. The framework is intended as a working hypothesis, not as a definitive explanation. Specifically, we hypothesize that, in a subset of patients with persistent, moderate-to-severe, type 2-high AR, the olfactory cleft neuroepithelium may undergo alarmin-driven type 2 inflammatory stress that contributes to persistent smell loss despite improvement or exclusion of macroscopic obstruction. The model integrates mechanistically plausible inferences from murine olfactory inflammation models, ex vivo olfactory preparations, human nasal mucosal immunology, and CRSwNP biology. Importantly, CRSwNP evidence is used only as a mechanistic analogue and not as direct clinical evidence for AR-OD. The model is not intended to explain all AR-associated smell loss. In patients with mild or intermittent AR, transient mucosal edema and conductive odorant-access limitation may remain the dominant mechanism. Our aim is to articulate a coherent, falsifiable hypothesis and to define a stepwise experimental path toward its validation, refinement, or refutation.

### 1.4 Central evidence gap and evidence hierarchy

**Figure 1:**
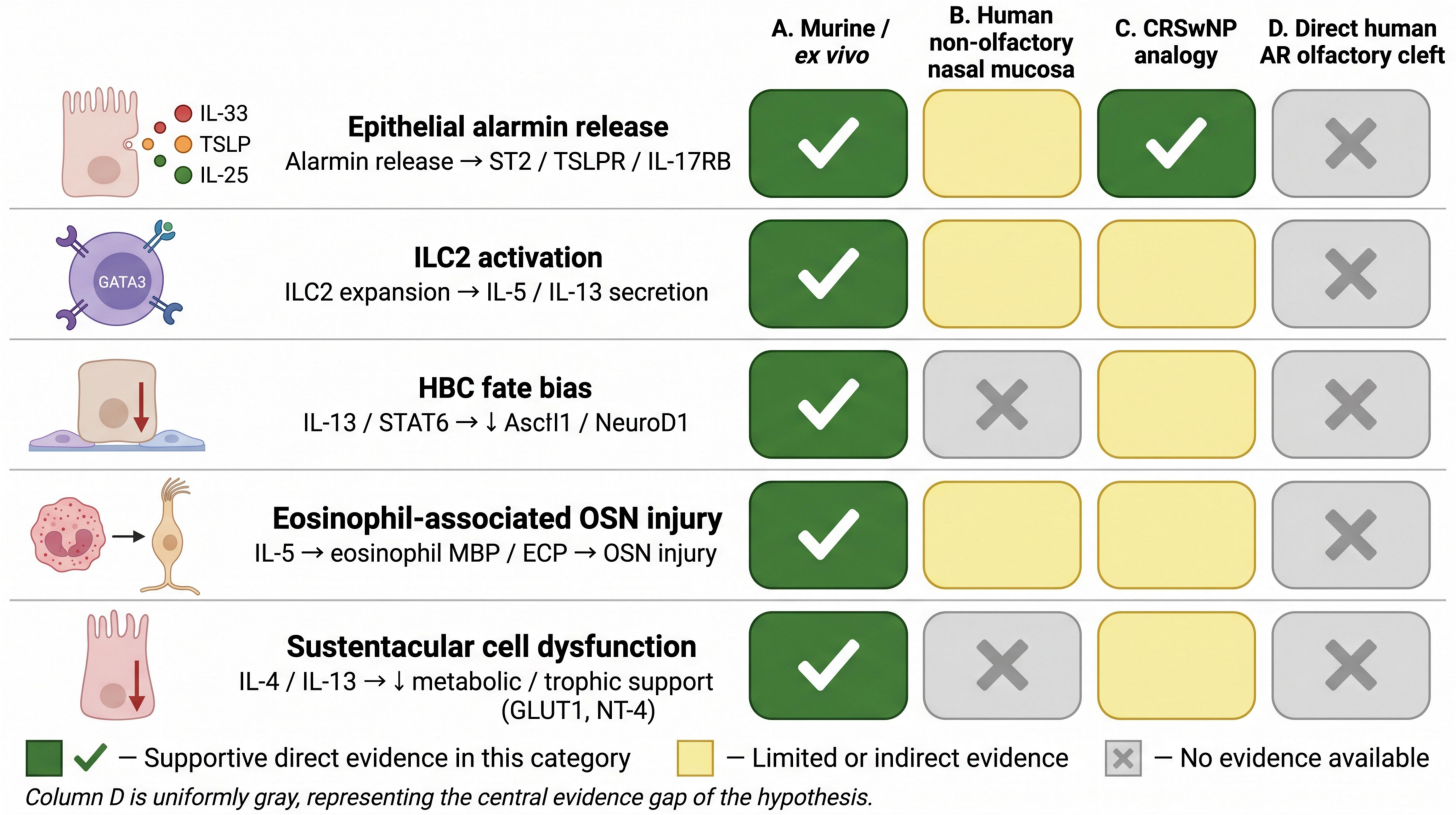
Evidence-level map for the five candidate components of the alarmin–ILC2 hypothesis in AR-OD. Each row depicts one proposed mechanistic component with a small cellular icon and its key signaling molecule. To the right, four colored evidence cells indicate the highest currently available evidence level in each category (murine/ex vivo, human non-olfactory nasal mucosa, CRSwNP analogy, direct human AR olfactory cleft). Green = supportive direct evidence; yellow = indirect or limited evidence; gray with ✗ = no evidence. The rightmost column is uniformly gray, emphasizing the central evidence gap motivating this hypothesis. AR-OD, AR-associated olfactory dysfunction; CRSwNP, chronic rhinosinusitis with nasal polyps; HBC, horizontal basal cell; IL, interleukin; ILC2, group 2 innate lymphoid cell; OSN, olfactory sensory neuron; SC, sustentacular cell; TSLP, thymic stromal lymphopoietin.

The central limitation of the present hypothesis is that none of the proposed mechanistic steps has yet been directly validated in human AR olfactory cleft tissue. Specifically, no study has directly demonstrated olfactory cleft alarmin release, ILC2 enrichment, horizontal basal cell (HBC) fate bias, eosinophil-mediated OSN injury, or sustentacular cell dysfunction in patients with AR-associated OD. We therefore classify the evidence used in this article into four levels: Level A: direct evidence from human AR olfactory cleft tissue; Level B: human AR evidence from non-olfactory nasal mucosa or nasal secretions; Level C: evidence from CRS/CRSwNP used as mechanistic analogy; Level D: evidence from murine models, ex vivo systems, organoids, or general airway immunology. At present, all proposed downstream injury modules lack Level A evidence. The model is therefore presented as a candidate, falsifiable hypothesis rather than a validated causal pathway (**Figure 1**).

## 2 The proposed alarmin–ILC2/type 2 neuroimmune hypothesis

### 2.1 The olfactory cleft as a candidate vulnerable neuroimmune niche

The olfactory epithelium (OE) occupies a specialized region along the superior nasal vault and upper septum and provides the peripheral sensory substrate for olfaction (8, 9). Unlike the respiratory epithelium (RE), which contains a ciliated architecture supporting mucociliary clearance, the OE is covered by a relatively thin mucus layer and contains OSN dendritic knobs and cilia exposed to inhaled odorants and environmental agents within Bowman’s gland-derived secretions (8, 10). This organization enables sensory detection but may also increase vulnerability to local epithelial stress and inflammatory injury.

Three epithelial cell populations are particularly relevant to this hypothesis. OSNs detect odorants through apical cilia and project axons to the olfactory bulb (8, 9). Sustentacular cells provide metabolic, detoxifying, ionic, and trophic support to OSNs (8, 10). Microvillar cells constitute another epithelial population projecting into the mucus layer. Recent single-cell studies suggest that at least a subset of these cells share tuft-cell-like transcriptional and chemosensory features, including potential involvement in IL-25 and lipid mediator signaling (11–13). We therefore refer to them cautiously as tuft-like microvillar cells rather than assuming full equivalence to intestinal tuft cells in human AR.

The basal compartment contains globose basal cells (GBCs), which support constitutive OSN turnover, and horizontal basal cells (HBCs), which act as reserve progenitors after more severe epithelial injury (14, 15). The balance between ongoing GBC-mediated neurogenesis and injury-induced HBC activation is central to olfactory epithelial repair. We hypothesize that persistent type 2 inflammation may perturb this regenerative balance in a subset of AR patients with persistent OD.

The OE also harbors resident immune cells. Single-cell RNA sequencing data indicate that sustentacular cells express innate immune pattern recognition receptors, including TLR2, TLR4, and components of the nuclear factor kappa-B (NF-κB) signaling pathway (8, 10). ILC2s have been identified in human nasal mucosa, and studies of AR have detected lineage-negative, GATA3-positive or CRTH2-positive ILC2-like populations predominantly in inferior turbinate or other non-olfactory nasal mucosal samples (16, 17). These findings show that allergic nasal mucosa can support ILC2 accumulation, but they do not establish ILC2 enrichment within the olfactory cleft. Direct sampling of functional olfactory cleft neuroepithelium is technically challenging and ethically constrained because of the theoretical risk to residual olfactory function. Consequently, no study has yet correlated olfactory cleft ILC2 abundance with objective TDI or UPSIT scores in AR.

Three anatomical features provide a plausible, but unproven, rationale for local alarmin accumulation in the olfactory cleft. First, compared with ciliated respiratory epithelium, the OE has more limited mucociliary clearance, which may prolong contact between deposited allergens and epithelial sensory surfaces. Second, the relatively thin Bowman’s gland-derived mucus layer may provide less dilutional capacity for locally released alarmins. Third, the narrow geometry of the olfactory cleft may permit local cytokine accumulation. These features should be interpreted as anatomical inferences rather than demonstrated determinants of ILC2 localization. They generate a testable prediction: persistent type 2-high AR-OD should be associated with stronger olfactory cleft alarmin and ILC2-associated signatures than AR without OD (8–10).

### 2.2 Epithelial alarmin release as a candidate upstream event

We propose that, in susceptible patients with persistent type 2-high AR, protease-active aeroallergens such as house dust mite or mold components may induce epithelial barrier stress in the olfactory cleft. In airway epithelial systems, allergen proteases can disrupt tight-junction and adherens-junction integrity, including proteins such as occludin and E-cadherin. Whether the same process occurs in human AR olfactory cleft epithelium remains to be directly tested.

Based on airway mucosal biology, epithelial stress could promote rapid extracellular release of preformed nuclear IL-33 from injured epithelial or sustentacular cell populations and induce TSLP transcription and secretion over subsequent hours (18–21). In the proposed AR-OD model, these events represent candidate upstream signals rather than established features of human AR olfactory cleft tissue.

IL-25 may represent a complementary alarmin signal in the olfactory region. Single-cell transcriptomic studies of murine and human nasal mucosa have identified tuft-like microvillar cell populations within the olfactory region that express tuft-cell-associated genes such as Trpm5, Pou2f3, Chat, and Avil, as well as Il25 and eicosanoid biosynthetic enzymes including Alox5, Alox5ap, and Ltc4s (11). In murine olfactory mucosa, TRPM5-positive tuft-like microvillar cells were reported to be enriched relative to some other airway sites, suggesting that the olfactory region may contain a local IL-25 and lipid mediator source (11). Complementary airway studies indicate that tuft-cell-derived IL-25 and cysteinyl leukotrienes (CysLTs) can synergize to promote ILC2 activation and eosinophilic inflammation (12, 13). These findings support the plausibility of an IL-25–CysLT–ILC2 circuit, but whether an analogous circuit operates in the human AR olfactory cleft remains unknown.

In experimental airway inflammation models, upstream epithelial alarmins can cooperate to amplify type 2 inflammation (18, 19). In patients with AR, IL-33 and TSLP have been detected in nasal lavage during allergen challenge, but available samples have not been specific to the olfactory cleft (20). Therefore, a central biomarker prediction of this article is that olfactory cleft-specific IL-33, TSLP, IL-25, IL-13, and IL-5 profiles should differ between persistent AR patients with and without objective OD.

### 2.3 ILC2s as candidate early amplifiers within a broader type 2 network

ILC2s express receptors for epithelial alarmins, including ST2 for IL-33, TSLPR for TSLP, and IL-17RB for IL-25, and can produce type 2 cytokines after alarmin stimulation through GATA3-dependent programs and associated intracellular signaling pathways (22–24). Because ILC2 activation does not require antigen-specific T-cell receptor rearrangement or classical antigen presentation, ILC2s may respond rapidly to epithelial stress signals. This property provides a plausible basis for considering ILC2s as early amplifiers of type 2 inflammation after allergen exposure. However, in the present AR-OD model, ILC2s are not proposed as the sole source of IL-5 or IL-13, but as one component of a broader network that also includes mast cells, basophils, dendritic cells, and memory Th2 cells.

**Figure 2:**
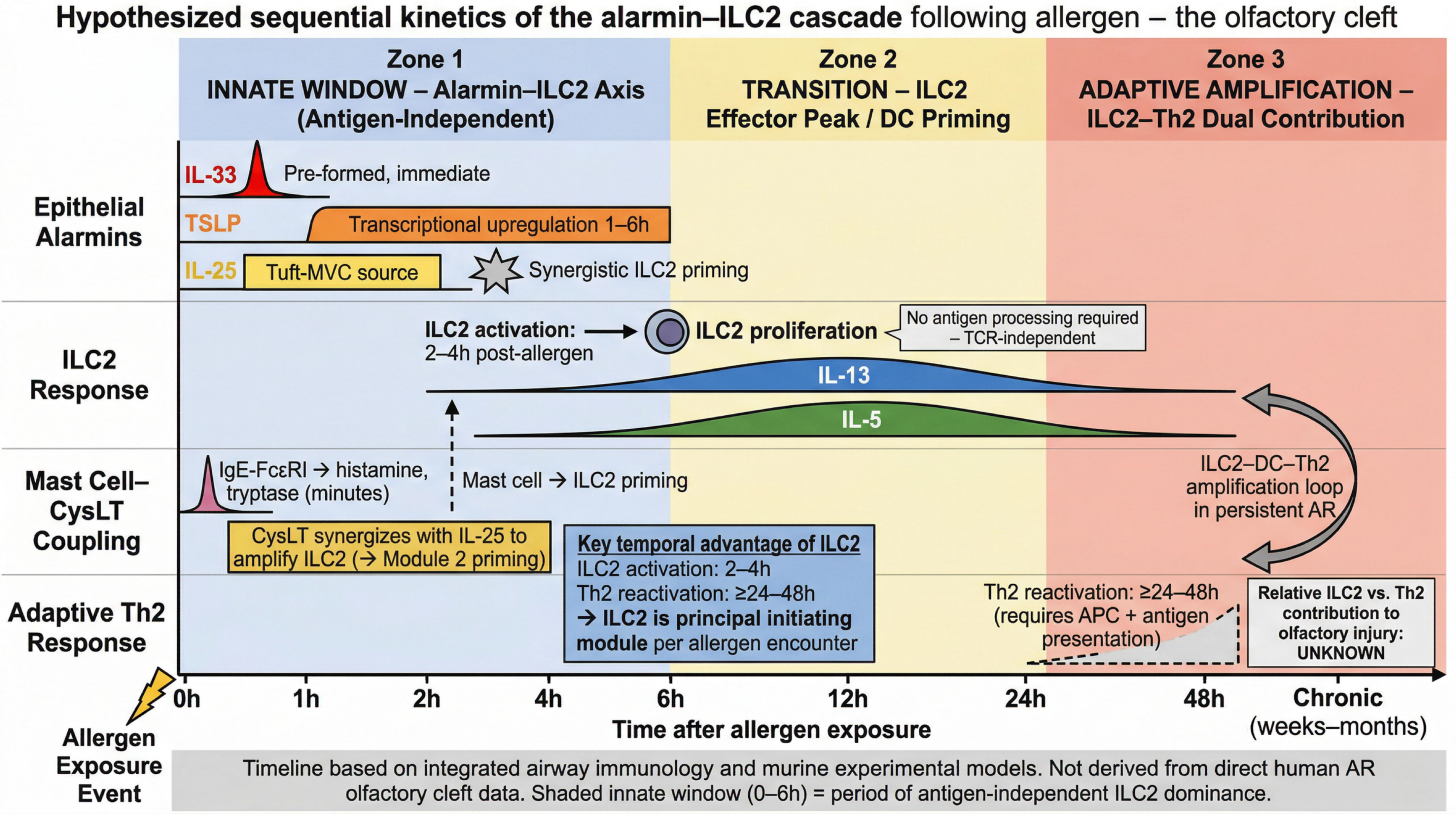
Proposed temporal network linking early epithelial alarmins, ILC2 activation, and adaptive type 2 amplification after allergen exposure. This conceptual timeline illustrates a proposed, unvalidated sequence after allergen exposure. During the immediate phase, IgE-dependent mast cell activation may release histamine, tryptase, CysLTs, and prostaglandin D_2_, which can influence vascular permeability, epithelial stress, and potentially OSN signaling. In parallel, epithelial injury may release IL-33 and induce TSLP, while tuft-like microvillar cells may provide IL-25 and lipid mediator signals. During the early alarmin-responsive phase, ILC2s may amplify type 2 cytokine production. With repeated or chronic allergen exposure, dendritic cells, basophils, memory Th2 cells, mast cells, and ILC2s likely form a co-sustaining type 2 inflammatory network rather than a strictly sequential cascade. The time windows shown are inferential and derived from airway immunology and murine models; they should not be interpreted as established kinetics in human AR olfactory cleft tissue. CysLT, cysteinyl leukotriene; DC, dendritic cell; HBC, horizontal basal cell; IgE, immunoglobulin E; ILC2, group 2 innate lymphoid cell; OSN, olfactory sensory neuron; SC, sustentacular cell; ST2, IL-33 receptor; Th2, type 2 helper T cell; TSLP, thymic stromal lymphopoietin; tuft-MVC, tuft-like microvillar cell.

This temporal model is most applicable to early or acute allergen exposure. In chronic persistent AR, the inflammatory state is unlikely to be strictly sequential. Instead, epithelial alarmins, IgE-dependent mast cell activation, basophils, dendritic cells, ILC2s, and allergen-specific memory Th2 cells probably form a self-amplifying type 2 network (25). ILC2s may dominate some early alarmin-responsive events, whereas Th2 cells and IgE-dependent effector cells may become co-dominant during chronic disease. The relative contributions of ILC2-derived versus Th2-derived IL-13 and IL-5 in the human AR olfactory cleft remain unknown and represent a key testable question (**Figure 2**).

Human nasal mucosal studies have identified ILC2-related signatures in eosinophilic inflammatory states (17). Whether comparable ILC2-like or type 2 immune niches are enriched specifically in the olfactory cleft of persistent AR-OD patients remains untested. This is a spatial prediction of the present hypothesis rather than an established observation.

### 2.4 Three candidate downstream injury modules

**Figure 3:**
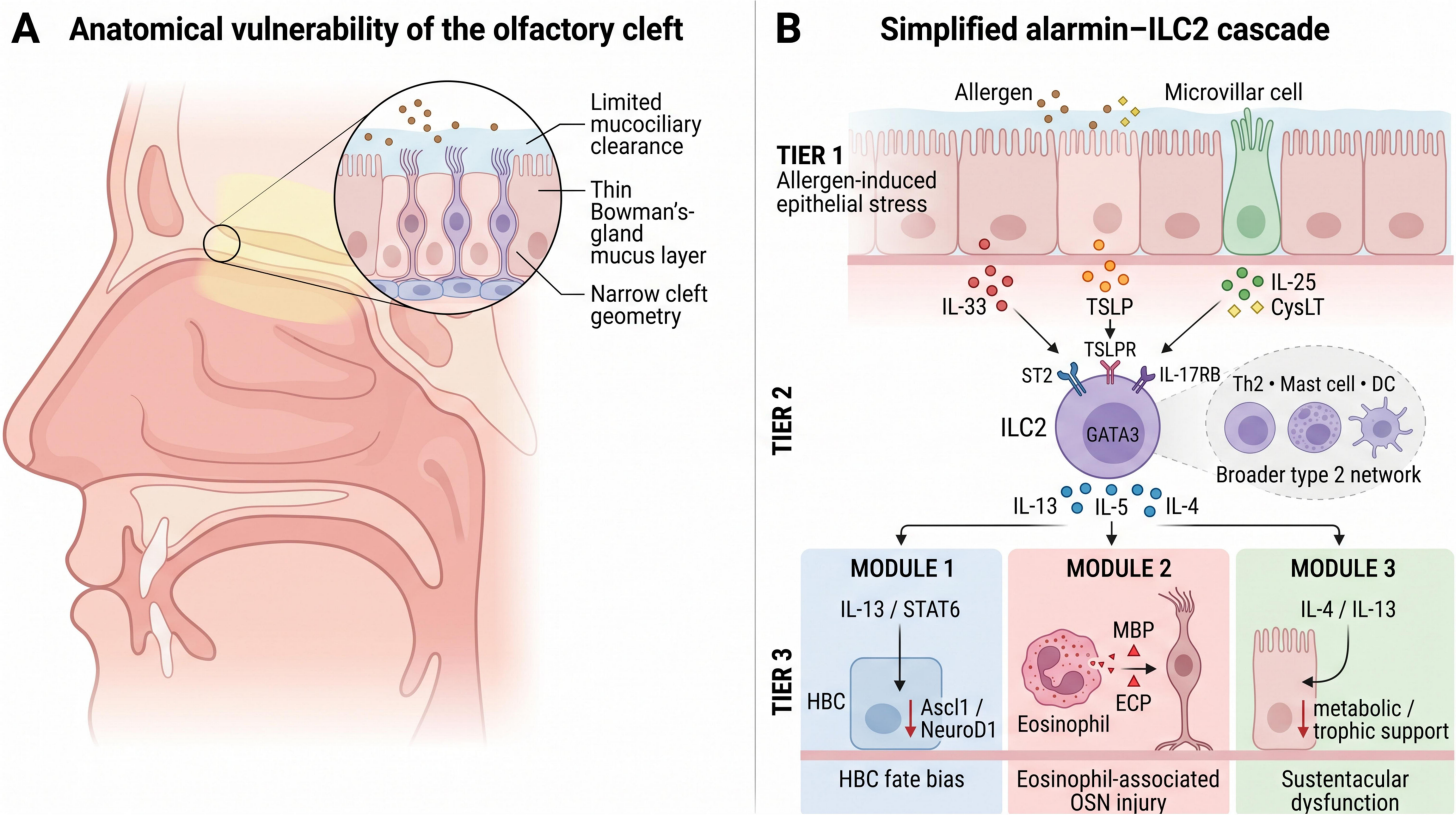
Simplified hypothesis model of olfactory cleft vulnerability and unvalidated candidate type 2 neuroimmune injury in persistent AR-OD. **(A)** shows a mid-sagittal view of the human nasal cavity highlighting three anatomical features of the olfactory cleft that may favor local epithelial stress: limited mucociliary clearance, a thin Bowman’s gland–derived mucus layer, and narrow cleft geometry. **(B)** presents a simplified cellular cascade: allergen-induced epithelial stress releases IL-33 and TSLP from sustentacular/epithelial cells, while tuft-like microvillar cells contribute IL-25 and lipid mediator signals. These alarmins engage ST2, TSLPR and IL-17RB on ILC2s, which act as early amplifiers within a broader type 2 network including Th2 cells and mast cells. Three candidate downstream injury modules are depicted: IL-13/STAT6 → HBC fate bias; IL-5/eosinophil → OSN injury via MBP/ECP; IL-4/IL-13 → sustentacular cell dysfunction. None of the pathways shown has been directly validated in human AR olfactory cleft tissue. AR-OD, AR-associated olfactory dysfunction; ECP, eosinophil cationic protein; HBC, horizontal basal cell; IL, interleukin; IL-17RB, interleukin-17 receptor B; ILC2, group 2 innate lymphoid cell; MBP, major basic protein; OSN, olfactory sensory neuron; STAT6, signal transducer and activator of transcription 6; ST2, IL-33 receptor (IL1RL1); Th2, type 2 helper T cell; TSLP, thymic stromal lymphopoietin; TSLPR, TSLP receptor.

We organize the possible sensorineural consequences of olfactory cleft type 2 inflammation into three candidate downstream injury modules. These modules are conceptually distinct but may overlap biologically. Current support derives mainly from murine systems, ex vivo preparations, human non-olfactory nasal mucosal studies, and CRSwNP studies used only as mechanistic analogies. None has been directly demonstrated in human AR olfactory cleft tissue (**Figure 3**).

#### 2.4.1 Module 1: IL-13/STAT6-associated basal cell fate bias

In a murine model of chronic olfactory IL-13 overexpression, *Saraswathula* et al. reported progressive, regionally specific OSN loss and HBC morphological changes consistent with aberrant activation without productive neuronal output (26). These findings suggest that sustained IL-13 signaling can perturb olfactory epithelial homeostasis in mice. In the proposed AR-OD model, IL-13 acting through the IL-13Rα1/IL-4Rα receptor complex and STAT6 signaling is hypothesized to bias HBC fate away from productive neurogenesis, potentially suppressing the Ascl1–Neurogenin1–NeuroD1 cascade. Separately, *Chen* et al. showed that chronic murine olfactory inflammation can induce an NF-κB-dependent HBC functional switch toward immune-defense programs, including CCL19 and CCL20 expression, at the expense of neuronal regeneration (27). This mechanism is conceptually relevant but was not originally defined in an allergen-driven type 2 AR model. Thus, NF-κB-mediated HBC immune switching should be considered a parallel mechanism that may converge with, but has not been proven to be downstream of, the alarmin–ILC2 axis in AR. Whether IL-13/STAT6-associated HBC fate bias occurs in human AR olfactory cleft tissue remains unknown.

#### 2.4.2 Module 2: IL-5/eosinophil-associated OSN injury

IL-5 is a central cytokine regulating eosinophil maturation, survival, and tissue recruitment. In the olfactory cleft, locally increased IL-5 derived from ILC2s and/or Th2 cells could promote eosinophil accumulation and degranulation. Degranulating eosinophils release major basic protein (MBP) and eosinophil cationic protein (ECP), which can damage epithelial and neuronal membranes. In experimental systems, eosinophilic inflammation has been associated with OSN apoptosis, olfactory cilia loss, and reduced electroolfactogram responses (28, 29).

*Kim* et al. reported in murine AR models that IL-5, together with TNF-α, can induce apoptosis in olfactory sphere cells and impair regenerative output (28). Human correlates are suggested by studies linking eosinophilic inflammation with impaired olfactory signal-transduction-associated markers or smell impairment in AR (3, 7).

#### 2.4.3 Module 3: IL-4/IL-13-associated sustentacular cell dysfunction

Sustentacular cells provide metabolic, detoxifying, ionic, and trophic support to OSNs (8, 10). In murine IL-13 overexpression models, IL-13 signaling through IL-13Rα1 on sustentacular cells alters inflammatory and xenobiotic metabolism-associated pathways, including Ym2/Chil4-linked programs (26, 30). These changes suggest that type 2 cytokines may modify the supportive niche required for OSN survival and repair.

*Hara* et al. further reported that IL-4 can impair OSN odorant responsiveness ex vivo and that this effect is reversible by IL-4Rα blockade (31). Together, these findings lead us to hypothesize that IL-4/IL-13 signaling may weaken sustentacular support functions and reduce OSN resilience to inflammatory stress. This module remains speculative in human AR because direct sustentacular cell profiling from AR olfactory cleft tissue has not yet been performed. The core features and evidence levels of these candidate modules are summarized in **Table 2**.

**Table 2:** Summary of candidate mechanistic modules and highest currently available evidence level.

Candidate module	Key cytokine(s)	Target cell	Predicted consequence	Highest evidence level
Alarmin-driven ILC2 activation	IL-33, TSLP, IL-25	ILC2	Type 2 cytokine amplification	Murine + human inferior turbinate (Levels B/D)
HBC fate bias	IL-13	HBC	Impaired neurogenic trajectory	Murine IL-13 overexpression (Level D)
Eosinophilic OSN injury	IL-5	Eosinophils → OSN	OSN apoptosis, cilia loss, reduced EOG	Murine AR models (Level D)
Sustentacular cell dysfunction	IL-4, IL-13	Sustentacular cell	Reduced metabolic/trophic support	Murine + ex vivo OSN preparations (Level D)
HBC immune switch (parallel)	NF-κB ligands (TNF-α, etc.)	HBC	Immune-defense phenotype at expense of neurogenesis	Murine non-type 2 inflammation (Level D)

This condensed table is intended only as a quick reference. None of the proposed mechanisms has been directly demonstrated in human AR olfactory cleft tissue, which constitutes the central evidence gap of the hypothesis. Evidence levels follow the hierarchy defined in Section 1.4. Level A (direct human AR olfactory cleft) evidence is absent for all modules. Evidence levels follow the hierarchy defined in Section 1.4. Detailed receptor/signaling annotations (STAT6, JAK2/STAT5, MAPK/ERK, PI3K/AKT, MBP/ECP, Ym2/Chil4, etc.). AR, allergic rhinitis; AR-OD, AR-associated olfactory dysfunction; EOG, electroolfactogram; HBC, horizontal basal cell; IL, interleukin; ILC2, group 2 innate lymphoid cell; NF-κB, nuclear factor kappa-B; OSN, olfactory sensory neuron; STAT6, signal transducer and activator of transcription 6; TNF-α, tumor necrosis factor alpha; TSLP, thymic stromal lymphopoietin.

## 3 Evidence supporting the hypothesis

### 3.1 Human clinical observations consistent with a sensorineural component

The strongest human evidence currently available and consistent with a sensorineural component in AR-associated OD comes from clinical studies showing partial dissociation between congestion severity and olfactory performance. Stuck and Hummel reported variable and incomplete olfactory improvement after INCS in AR (5). *Guilemany* et al. found that smell loss in persistent AR related not only to congestion but also to local inflammatory features (6). *Fornazieri* et al. documented a substantial prevalence of objective OD in AR and reported altered olfactory signal-transduction-associated markers in eosinophilic disease (3). *Klimek* and *Eggers* found an association between nasal eosinophilia and olfactory impairment (7). *Wu* et al. reported that olfactory cleft and middle meatal cytokine levels correlate with smell function in CRS; however, this evidence is not AR-specific and should be interpreted only as indirect support (32).

However, no published study has directly measured IL-33, TSLP, IL-13, IL-25, IL-5, ILC2 abundance, HBC transcriptional state, OSN loss, or sustentacular cell dysfunction specifically in the olfactory cleft of patients with AR and correlated these variables with objective TDI or UPSIT scores. Human AR ILC2 data remain largely restricted to inferior turbinate or non-olfactory nasal mucosal samples (16). This absence of Level A evidence constitutes the central evidence gap of the present hypothesis.

### 3.2 Indirect mechanistic evidence from murine and ex vivo studies

Mechanistic evidence relevant to the hypothesis derives from murine and ex vivo systems. *Saraswathula* et al. reported that sustained olfactory mucosal IL-13 overexpression in mice leads to regional OSN depletion and HBC changes consistent with impaired regenerative output (26). *Chen* et al. showed that chronic murine olfactory inflammation can induce an NF-κB-dependent HBC switch from neuroregeneration toward immune defense (27). *Wang* et al. linked Ym2/Chil4-associated inflammatory pathways to olfactory epithelial regeneration (30). *Kim* et al. reported that IL-5 and TNF-α induce apoptosis in murine olfactory sphere cells, impairing regenerative replenishment (28). *Liang* et al. developed a murine AR-associated olfactory loss model linking chronic allergic inflammation to permanent OSN depletion (29).

These studies support the biological plausibility of inflammatory injury to the olfactory neuroepithelium, but they do not establish that the same sequence occurs in human AR olfactory cleft tissue. Species-specific constraints on extrapolation are addressed in **Supplementary Table 1**.

### 3.3 CRSwNP data as indirect rationale, not validation for AR-OD

CRSwNP and AR share several type 2 inflammatory features, including epithelial alarmin signaling, ILC2/Th2 activation, eosinophilic inflammation, and IL-4/IL-13/IL-5 effector pathways. However, CRSwNP differs fundamentally from AR without polyps because it involves macroscopic polypoid remodeling, chronic tissue architectural distortion, and frequent olfactory cleft obstruction. Therefore, improvement in smell after biologic therapy in CRSwNP may reflect reduced obstruction, reduced inflammation, sensorineural recovery, or a combination of these mechanisms.

For this reason, CRSwNP biologic trials are used here only as indirect rationale for hypothesis generation, not as evidence that the same therapeutic hierarchy applies to AR-OD. Dupilumab, tezepelumab, and other biologics have improved smell-related outcomes in CRSwNP trials, and ex vivo or murine studies suggest that IL-4/IL-13 and upstream alarmin pathways can affect olfactory neuroepithelial function (31, 33–38). These observations are consistent with the possibility that type 2 inflammatory pathways influence olfactory function in sinonasal disease. However, they do not validate the alarmin–ILC2 hypothesis in AR-OD and do not establish a therapeutic hierarchy for AR-associated smell loss.

The CATNIP trial further suggests that upstream alarmin modulation can reshape allergen-induced nasal epithelial type 2 responses, although it was not an olfactory study and was not specific to the olfactory cleft (39). Dedicated studies in AR patients without nasal polyps and without macroscopic olfactory cleft obstruction are required to determine whether similar pathways operate in AR-OD.

## 4 Alternative explanations and limitations

### 4.1 Conductive and sensorineural mechanisms are not mutually exclusive

The present hypothesis is intended to complement, rather than replace, the conductive obstruction model. AR-associated OD is likely mechanistically heterogeneous. In patients with intermittent or mild AR, mucosal edema and transient olfactory cleft narrowing may be the dominant mechanism, and improvement in nasal airflow may substantially restore smell. In persistent moderate-to-severe AR, cumulative allergen exposure may engage additional inflammatory mechanisms that could contribute to microscopic neuroepithelial vulnerability. Such changes are predicted to include impaired basal cell neurogenesis, reduced OSN resilience, and sustentacular cell dysfunction, but these events have not yet been demonstrated in human AR olfactory cleft tissue. The relative contribution of conductive and sensorineural mechanisms is likely patient-specific and remains an important question for future studies (40).

### 4.2 Disease heterogeneity in allergic rhinitis

AR is not a biologically uniform condition. The proposed alarmin–ILC2/type 2 neuroimmune hypothesis is most relevant to patients with persistent, moderate-to-severe, eosinophil-dominant or type 2-high AR, especially when objective OD is disproportionate to measured nasal obstruction. Important modifiers include allergen sensitization pattern, perennial versus seasonal exposure, Allergic Rhinitis and its Impact on Asthma (ARIA) severity category, asthma comorbidity, blood and local eosinophil burden, local allergic rhinitis, prior immunotherapy, and recent corticosteroid or antihistamine use. The model should not be generalized to all AR phenotypes.

### 4.3 Other plausible immune and neural contributors

The alarmin–ILC2 axis is proposed as one candidate pathway within a broader allergic neuroimmune network, not as the sole or dominant driver of AR-associated OD in all patients. IgE-dependent mast cell activation, basophils, dendritic cells, adaptive Th2 cells, eosinophils, epithelial barrier dysfunction, vascular changes, neurogenic inflammation, and conductive obstruction may all contribute to smell loss in different patients.

Mast cell-mediated immediate-phase responses should be viewed as complementary rather than subordinate to the alarmin–ILC2 hypothesis. In sensitized AR, allergen engagement of IgE–FcϵRI complexes on mast cells can rapidly release histamine, tryptase, CysLTs, and prostaglandin D_2_. These mediators may influence nasal airflow, epithelial stress, vascular permeability, and possibly OSN responsiveness. CysLTs and prostaglandin D_2_ can also promote ILC2 activation in airway models, suggesting a potential bridge between IgE-dependent immediate responses and alarmin-responsive innate type 2 amplification. However, the strength, timing, and olfactory relevance of this mast cell–ILC2 coupling in the human AR olfactory cleft remain unknown (41).

Additional mechanisms that merit consideration include IgE-mediated pathways. CRSwNP data comparing IL-4Rα blockade and IgE blockade raise a testable question about the relative contribution of IL-4/IL-13-dependent versus IgE-dependent pathways in type 2 inflammatory smell loss, but these findings cannot be directly extrapolated to AR-OD (35). As reported by *Hara* et al., the direct effect of IL-4 on OSN odorant responsiveness also warrants further consideration (31, 35)Other plausible contributors include neuropeptide-mediated neurogenic inflammation involving substance P and calcitonin gene-related peptide (CGRP). Furthermore, bidirectional neuro-immune crosstalk likely plays a key role. In this process, damaged OSNs may release damage-associated molecular patterns (DAMPs) that reciprocally amplify ILC2 activation or suppress HBC neurogenesis. This potential feedback loop—if it exists—would expose an additional vulnerability by maintaining a self-reinforcing cycle of olfactory injury.

*Li* et al. reported that chronic intranasal corticosteroid exposure can induce OSN degeneration in murine models (42). The clinical relevance of this finding to human AR treatment remains uncertain, but it highlights the need to distinguish beneficial anti-inflammatory effects from potential effects on olfactory epithelial homeostasis in experimental studies.

### 4.4 Species-specific constraints on extrapolation

Most mechanistic evidence informing the hypothesis derives from OVA- or HDM-sensitized murine models, IL-13 overexpression systems, ex vivo preparations, and general airway immunology. These systems differ from human AR in allergen exposure pattern, olfactory epithelial architecture, OSN receptor repertoire, inflammatory kinetics, immune-cell composition, and regenerative capacity (43). These differences do not invalidate the hypothesis but require that each proposed mechanism be independently tested in human AR olfactory cleft or ethically available adjacent tissue. Detailed species-specific constraints are summarized in **Supplementary Table 1**.

## 5 Evidence gaps and falsifiable predictions

The value of this hypothesis lies in its falsifiability. Because direct human AR olfactory cleft evidence is currently absent, each proposed mechanism should be treated as a prediction to be tested rather than as a conclusion. The hypothesis generates five major falsifiable predictions.

First, olfactory cleft mucus from persistent type 2-high AR-OD patients should show higher alarmin and type 2 effector signatures than mucus from AR patients without OD after adjustment for obstruction and confounders. Second, ILC2-like or type 2 immune niches should be detectable in olfactory cleft or adjacent olfactory mucosal samples and should spatially relate to alarmin-expressing epithelial regions. Third, OD-positive AR samples should show basal-cell transcriptional changes consistent with impaired neurogenic trajectories. Fourth, OSN injury or sustentacular dysfunction markers should associate with local type 2 biomarkers and objective olfactory scores. Fifth, in interventional studies, improvement in TDI or UPSIT should track more closely with reduction of local type 2 biomarkers than with airflow improvement alone. Findings that would weaken each prediction are summarized in **Table 3**.

**Table 3:** Major evidence gaps, falsifiable predictions, and findings that would weaken the alarmin–ILC2 hypothesis in AR-OD.

Evidence gap	Prediction if the hypothesis is correct	Finding that would weaken the hypothesis
No olfactory cleft-specific alarmin data in AR-OD	Persistent type 2-high AR-OD will show higher olfactory cleft IL-33, TSLP, IL-25, IL-13, IL-5, and eosinophil granule proteins than AR without OD	No biomarker difference between AR-OD and AR without OD after adjustment for obstruction and confounders
No direct ILC2 localization data in human AR olfactory cleft	ILC2-like or type 2 immune niches will be enriched near alarmin-expressing epithelial regions	ILC2-like cells are absent or rare in olfactory cleft samples
No HBC/GBC fate data in AR-OD	OD-positive AR samples will show increased pSTAT6-associated basal cell changes and reduced ASCL1/NEUROD1-positive neurogenic trajectories	Basal cell transcriptional states do not differ by OD status
No direct OSN injury data	OD-positive AR samples will show reduced OSN markers or increased eosinophil-associated injury signatures	OSN markers do not associate with type 2 biomarkers or smell scores
No sustentacular cell data	OD-positive AR samples will show altered sustentacular metabolic/trophic markers such as CYP-related genes, GLUT1, or neurotrophin-associated pathways	Sustentacular signatures remain normal despite type 2 inflammation
No AR-OD biologic trial data	If type 2 sensorineural injury is dominant, biomarker reduction should track with TDI/UPSIT improvement more strongly than airflow improvement alone	Smell improvement tracks only with airflow or occurs without biomarker change

This table summarizes hypothesis-derived predictions and potential falsifying observations. The predictions are intended for well-phenotyped cohorts of persistent, moderate-to-severe, type 2-high AR-OD without nasal polyps or macroscopic olfactory cleft obstruction. A negative result in one row would weaken the corresponding component of the model but would not necessarily refute all inflammatory mechanisms of AR-associated OD. Consistently negative findings across multiple domains, especially when adjusted for obstruction and relevant confounders, would substantially challenge the alarmin–ILC2 hypothesis. AR, allergic rhinitis; AR-OD, AR-associated olfactory dysfunction; ASCL1, achaete-scute family bHLH transcription factor 1; GBC, globose basal cell; HBC, horizontal basal cell; IL, interleukin; ILC2, group 2 innate lymphoid cell; NEUROD1, neuronal differentiation 1; OD, olfactory dysfunction; OSN, olfactory sensory neuron; pSTAT6, phosphorylated signal transducer and activator of transcription 6; TDI, threshold–discrimination–identification composite score; TSLP, thymic stromal lymphopoietin; UPSIT, University of Pennsylvania Smell Identification Test.

Candidate biologic intervention nodes are summarized in **Supplementary Figure 1** and **Supplementary Table 2**. These predictions are intended only as future proof-of-mechanism concepts and should not be interpreted as current treatment recommendations.

A secondary clinical prediction is that patients with shorter duration of persistent AR-OD may show greater reversibility than those with long-standing disease, if early dysfunction reflects inflammatory or metabolic perturbation rather than fixed neuroepithelial remodeling. This prediction requires prospective validation because disease duration, allergen exposure burden, age, prior treatment, and comorbid asthma may confound reversibility.

## 6 Stepwise validation strategy

Because direct biopsy of functional olfactory neuroepithelium solely for research purposes raises technical and ethical concerns, validation should proceed stepwise. The most feasible approach is to begin with non-invasive or minimally invasive biomarker studies, followed by ethically available tissue analyses, patient-derived *in vitro* models, and only later biomarker-enriched interventional trials. Detailed experimental protocols are provided in **Supplementary Methods**.

### 6.1 Phase I: non-invasive phenotyping and olfactory cleft biomarkers

The first step should be a prospective observational study comparing three groups: persistent moderate-to-severe AR patients with objective OD, persistent AR patients without OD, and non-allergic controls. Objective smell testing should use validated instruments such as TDI or UPSIT. Macroscopic obstruction should be assessed using nasal endoscopy, olfactory cleft scoring, objective airflow measures, and CT when clinically indicated.

Olfactory cleft mucus could be collected using endoscopically guided microswabs, absorbent strips, or localized lavage. Candidate biomarkers should include IL-33, TSLP, IL-25, IL-13, IL-5, eosinophil granule proteins, local IgE where feasible, and epithelial or neuronal injury markers. Analyses should adjust for age, sex, season, allergen exposure, asthma, recent medication use, systemic type 2 biomarkers, and objective obstruction.

Because effect sizes for olfactory cleft alarmins in AR-OD are currently unknown, initial studies should be designed as exploratory pilot cohorts rather than definitive diagnostic studies. A pragmatic first-stage design could enroll approximately 30–50 participants per group to estimate biomarker variance, assess sampling reproducibility, and generate effect-size estimates for larger validation cohorts. Prespecified adjustment for obstruction, seasonality, allergen exposure, asthma, and recent medication use will be essential.

### 6.2 Phase II: ethically feasible tissue-based validation

Direct research-only biopsy of functional olfactory neuroepithelium should be approached cautiously because of the theoretical risk to smell function. Tissue-based validation should initially rely on ethically available samples, including olfactory cleft-adjacent mucosa obtained during clinically indicated endoscopic procedures in carefully consented participants. When true olfactory epithelium cannot be safely obtained, epithelial brushing, cytology, or mucus-based profiling should be prioritized. For available tissue, multiplex immunofluorescence, RNAscope, flow cytometry where feasible, and spatial transcriptomics could assess epithelial alarmin expression, ILC2-like cell localization, eosinophil infiltration, pSTAT6 signaling, HBC markers, GBC markers, OSN markers, and sustentacular cell metabolic markers.

### 6.3 Phase III: organoid and air–liquid interface models

Patient-derived olfactory epithelial organoids or air–liquid interface cultures may provide a safer and more controllable platform for mechanistic testing than repeated *in vivo* tissue sampling. These systems could be exposed to IL-33, TSLP, IL-25, IL-4, IL-13, IL-5, eosinophil-conditioned media, or combinations thereof. Candidate endpoints should include epithelial barrier integrity, pSTAT6 activation, ASCL1/NEUROD1 expression, OMP-positive neuronal differentiation, HBC activation state, sustentacular metabolic markers, neurotrophin expression, and OSN functional readouts where technically feasible.

### 6.4 Phase IV: biomarker-enriched interventional studies

Biologic intervention should be considered a later-stage proof-of-mechanism test rather than an immediate clinical recommendation. A phase II trial would be most informative if restricted to patients with persistent, moderate-to-severe, type 2-high AR-OD, objective hyposmia or anosmia, no nasal polyps, and no macroscopic olfactory cleft obstruction. Baseline olfactory cleft biomarkers should be used to enrich for patients with alarmin/type 2-high signatures. Potential designs include placebo-controlled add-on studies or randomized pathway-comparison studies. A comparison of IL-4Rα blockade and IgE blockade could test whether different type 2 pathways show distinct biomarker-linked olfactory effects in a biomarker-enriched subgroup. Primary olfactory endpoints should be change in TDI or UPSIT, while mechanistic endpoints should include olfactory cleft cytokine changes, eosinophil markers, objective nasal airflow, and olfactory cleft imaging.

Interventional studies should initially be powered as proof-of-mechanism trials rather than definitive efficacy trials. Small biomarker-enriched randomized studies may first test whether pathway blockade modifies olfactory cleft biomarkers and whether biomarker change correlates with TDI or UPSIT improvement. Larger efficacy trials should only be considered if these mechanistic endpoints are met.

### 6.5 Criteria that would weaken or refute the hypothesis

The hypothesis would be substantially weakened by any of the following findings in well-phenotyped AR-OD cohorts: 1) olfactory cleft alarmins or type 2 effector cytokines do not differ between AR patients with and without OD after adjustment for obstruction and confounders; 2) ILC2-like cells or type 2 immune niches are absent or extremely rare in olfactory cleft or adjacent olfactory mucosal samples; 3) HBC, GBC, OSN, or sustentacular cell abnormalities do not associate with local type 2 biomarkers; 4) biologic or pathway-directed reduction of local type 2 biomarkers does not improve objective smell scores; or 5) improvement in olfaction tracks entirely with nasal airflow rather than local biomarker changes. Equivalent olfactory improvement with IgE blockade and IL-4Rα blockade would not invalidate all inflammatory mechanisms, but it would weaken the proposed prioritization of IL-4/IL-13-dependent sensorineural injury.

## 7 Potential clinical and research implications If validated

If the alarmin–ILC2 hypothesis for AR-OD is validated through the experimental and interventional steps described above, several clinical implications would follow:

Biomarker-guided patient stratification. A non-invasive olfactory cleft biomarker panel could help identify AR patients at elevated risk for persistent or progressive OD before irreversible neuroepithelial remodeling occurs. If validated, such biomarkers could support closer olfactory monitoring, risk stratification, and enrollment into mechanism-guided clinical trials. Whether they should guide biologic therapy would require separate interventional evidence.

Rational biologic selection. The framework may provide a rationale for future biologic selection studies in refractory, biomarker-enriched AR-OD. For example, patients with dominant alarmin signatures might be candidates for future proof-of-mechanism studies of upstream blockade, whereas patients with strong IL-4/IL-13 or eosinophilic signatures might be appropriate for trials targeting those pathways. These implications remain investigational and should not be interpreted as current treatment recommendations.

Early intervention window hypothesis. Anti-inflammatory or pathway-targeted intervention initiated before fixed neuroepithelial remodeling may theoretically yield greater recovery than intervention after long-standing disease. However, the reversibility window is currently unknown and requires prospective validation.

Olfactory training as adjunctive therapy. If inflammatory injury contributes to AR-OD, reducing local inflammation might create a more permissive microenvironment for olfactory training and neuroplastic recovery. This potential synergy remains speculative and should be tested in controlled studies.

These clinical implications remain speculative until the hypothesis is directly validated in human AR olfactory tissue or ethically available adjacent tissue, and until prospective interventional trials confirm biomarker-linked olfactory benefit.

## 8 Conclusion

We propose that the alarmin–ILC2 axis may represent a candidate neuroimmune mechanism contributing to persistent or disproportionate olfactory dysfunction in a subset of patients with moderate-to-severe, type 2-high allergic rhinitis. This framework does not replace the conductive obstruction model, which remains highly relevant in many patients, particularly those with intermittent or congestion-dominant disease. Rather, it offers a complementary hypothesis for patients whose smell loss persists despite improvement or exclusion of macroscopic nasal obstruction.

The model integrates indirect observations from airway epithelial alarmin biology, ILC2 immunology, murine olfactory inflammation models, ex vivo olfactory preparations, human non-olfactory nasal mucosal studies, and CRSwNP studies used as mechanistic analogies. However, none of the proposed mechanisms—olfactory cleft alarmin release, ILC2 enrichment, HBC fate bias, eosinophil-mediated OSN injury, or sustentacular cell dysfunction—has been directly validated in human AR olfactory cleft tissue. This absence of direct evidence is the central limitation and the central motivation of the present hypothesis.

Accordingly, the value of the framework lies not in establishing causality at the present stage, but in defining a coherent and falsifiable research agenda. Future studies should begin with non-invasive olfactory cleft biomarker profiling, proceed to ethically feasible tissue and organoid-based validation, and ultimately test pathway modulation in biomarker-enriched AR-OD cohorts. Only through such stepwise validation can the proposed microscopic or molecular vulnerability of the olfactory cleft be confirmed, refuted, or refined.

## Data Availability

The original contributions presented in the study are included in the article/**Supplementary Material**. Further inquiries can be directed to the corresponding author/s.
