# Supplementary material for: The alarmin–ILC2 axis as a candidate mechanism for persistent olfactory dysfunction in allergic rhinitis
Source: Front Immunol. 2026 Jul 8;17:1867859. doi: 10.3389/fimmu.2026.1867859 (PMC13388388; doi:10.3389/fimmu.2026.1867859)
Supplement: Supplementary file 1 [file Supplementaryfile1.docx]

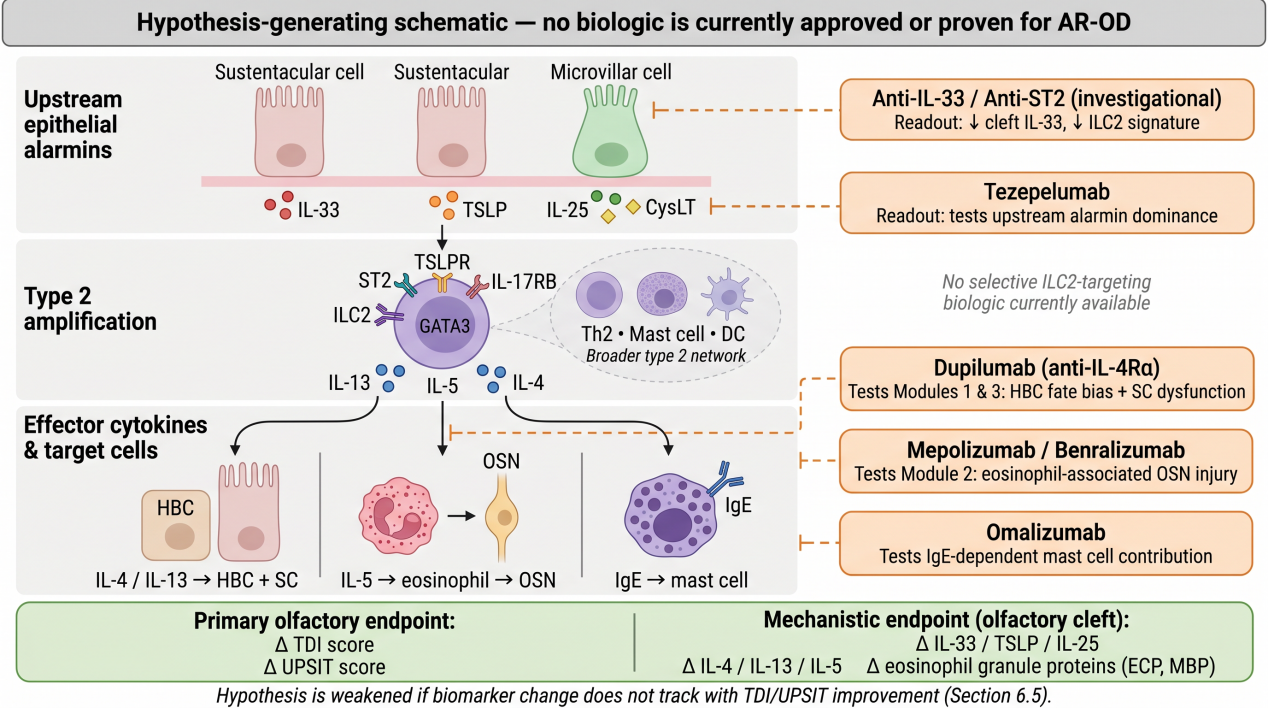


**Supplementary Figure S1. Candidate biologic intervention nodes for future proof-of-mechanism studies in biomarker-enriched AR-OD**

The schematic overlays five candidate biologic intervention nodes onto three tiers of the hypothesized cascade — upstream epithelial alarmins, ILC2/Th2 amplification, and downstream type 2 effectors. For each node, the candidate downstream injury module(s) tested and the predicted proof-of-mechanism readout are indicated. The bottom banner summarizes the dual endpoint structure proposed in Section 6.4. No biologic has been proven to improve olfactory dysfunction in AR-OD; clinical olfactory data for these agents derive mainly from CRSwNP or non-AR settings. Detailed predicted readouts and falsifying findings are provided in Supplementary Table S2.

Abbreviations: AR-OD, AR-associated olfactory dysfunction; CRSwNP, chronic rhinosinusitis with nasal polyps; IgE, immunoglobulin E; IL, interleukin; IL-4Rα, interleukin-4 receptor alpha; IL-5Rα, interleukin-5 receptor alpha; ILC2, group 2 innate lymphoid cell; OSN, olfactory sensory neuron; ST2, IL-33 receptor (IL1RL1); TDI, threshold–discrimination–identification composite score; Th2, type 2 helper T cell; TSLP, thymic stromal lymphopoietin; UPSIT, University of Pennsylvania Smell Identification Test.
