# Supplementary material for: The alarmin–ILC2 axis as a candidate mechanism for persistent olfactory dysfunction in allergic rhinitis
Source: Front Immunol. 2026 Jul 8;17:1867859. doi: 10.3389/fimmu.2026.1867859 (PMC13388388; doi:10.3389/fimmu.2026.1867859)
Supplement: Supplementary file 2 [file Table1.docx]

## Supplementary Table S1. Key translational constraints when extrapolating from murine allergic inflammation models to human AR-OD

| **Comparative Dimension** | **Murine OVA/HDM Model** | **Human AR** |
| --- | --- | --- |
| Sensitization/challenge paradigm | Intraperitoneal OVA sensitization or intranasal HDM; synchronized acute challenge | Natural, repeated aeroallergen inhalation; temporal variability in dose and interval |
| Olfactory epithelial architecture | Large OE surface area relative to nasal cavity; prominent zonal OSN receptor distribution | Restricted olfactory cleft OE (~2–4 cm² per side); less pronounced spatial zonation |
| OSN subtype composition | ~1,100 functional OR genes; distinct receptor expression patterns | ~400 functional OR genes; distinct OSN subtype distribution |
| ILC2 localization data | GATA3+ ILC2s detectable in murine OE in allergen challenge models | Human nasal ILC2 data restricted to inferior turbinate; olfactory cleft ILC2s not quantified |
| Type 2 inflammatory kinetics | Acute, synchronized eosinophilic infiltration following challenge | Episodic, cumulative; duration and chronicity vary substantially |
| T cell involvement | Th2 response well-characterized; limited Treg/Th17 counterregulation in acute models | Complex Th2/Treg/Th17/ILC2 interplay; modulated by immunotherapy history |
| HBC response to IL-13 | Morphological changes documented in transgenic IL-13 overexpression model | Direct IL-13 effects on human HBCs in AR context not demonstrated |
| Olfactory regenerative capacity | Active constitutive neurogenesis well-characterized; relatively rapid OSN turnover | Lower constitutive OSN turnover rate; regenerative capacity declines with age; human data sparse |

Table note: This table systematically enumerates the major interspecies differences in olfactory biology and type 2 immunology between murine AR models (OVA-sensitised and HDM-sensitised paradigms) and human AR, assessed across the following dimensions: sensitisation and challenge paradigm, olfactory epithelium anatomy, OSN subtypes and olfactory receptor (OR) gene composition, availability of ILC2 localisation data, type 2 inflammatory kinetics, T cell biology, HBC responses under IL-13 stimulation, and olfactory regenerative capacity. The ILC2 localisation gap (row 4) carries particular translational significance: all available human nasal ILC2 data are derived from inferior turbinate biopsies, which differ fundamentally from the olfactory cleft in epithelial architecture, mucus layer thickness, and allergen residence time. The hypothesis predicts that the olfactory cleft may favor ILC2 enrichment, and this prediction requires direct testing using the experimental designs described in Section 6.1. OR gene count estimates (approximately 1,100 in mice; approximately 400 functional genes in humans) are derived from current genomic annotation databases (*Saraiva et al.*). The lower basal OSN turnover rate in adult humans implies that inflammation-driven neurogenesis suppression produces a slower but more cumulative and less reversible OSN pool depletion than observed in murine models. These differences do not invalidate the value of the murine evidence base, but require independent verification of each mechanistic step in human AR olfactory cleft tissue before strong translational or therapeutic claims can be made.

Abbreviations: GATA3, GATA-binding protein 3; HBC, horizontal basal cell; HDM, house dust mite; ILC2, group 2 innate lymphoid cell; OR, olfactory receptor gene; OSN, olfactory sensory neuron; OVA, ovalbumin; Th2, type 2 helper T cell; Treg, regulatory T cell.

## Supplementary Table S2. Candidate intervention nodes for future proof-of-mechanism studies in biomarker-enriched AR-OD

| **Agent** | **Primary Target** | **Candidate module tested** | **Candidate proof-of-mechanism readout** | **Result that would weaken the prediction** |
| --- | --- | --- | --- | --- |
| Dupilumab | IL-4Rα; blocks IL-4/IL-13 signaling | Candidate Modules 1 and 3; possible direct IL-4 effect on OSNs | Reduction in IL-4/IL-13-linked biomarkers with parallel improvement in TDI/UPSIT would support involvement of IL-4/IL-13 pathways in biomarker-enriched type 2-high AR-OD | Lack of biomarker reduction or lack of association between biomarker change and olfactory improvement would weaken this pathway-specific prediction |
| Omalizumab | IgE | IgE-dependent mast cell activation | Improvement mainly in patients with strong IgE/mast cell signatures; may help test the contribution of IgE-dependent mast cell activation relative to local type 2 effector signatures | Comparable biomarker-linked olfactory response to IL-4Rα blockade would suggest that IgE-dependent mechanisms may contribute substantially in selected AR-OD subgroups |
| Tezepelumab | TSLP | Upstream epithelial alarmin signaling and ILC2/Th2 priming | Reduction in olfactory cleft TSLP/type 2 biomarkers with parallel smell improvement if TSLP is a dominant upstream signal | Biomarker reduction without objective olfactory improvement |
| Mepolizumab / Benralizumab | IL-5 / IL-5Rα | Candidate Module 2; eosinophilic OSN injury | Biomarker-linked improvement in eosinophil-high AR-OD would support an eosinophil-associated injury module in eosinophil-high AR-OD | Eosinophil depletion without olfactory benefit |
| Anti-IL-33 / Anti-ST2 agents | IL-33/ST2 signaling | Upstream epithelial alarmin signaling | Reduced ILC2/type 2 activation and biomarker reduction with olfactory improvement would support IL-33/ST2 involvement; absence of such association would weaken this node | Failure to suppress local type 2 activation or smell loss despite effective IL-33/ST2 blockade |

Table note: This table summarizes unvalidated, hypothesis-derived predictions and is not intended as a treatment recommendation. The dupilumab-versus-omalizumab comparison is proposed as one potential mechanistic test because IL-4Rα blockade targets IL-4/IL-13 signaling, whereas IgE blockade primarily targets IgE-dependent mast cell activation. However, the prediction of differential biomarker-linked olfactory response with IL-4Rα blockade in AR-OD is inferred from mechanism and CRSwNP analogies and has not been directly tested in AR-OD. All proposed trials should enroll biomarker-enriched patients with persistent, moderate-to-severe AR-OD, exclude nasal polyps and macroscopic olfactory cleft obstruction, and include objective nasal airflow measures to distinguish conductive from sensorineural improvement.

Abbreviations: AR-OD, AR-associated olfactory dysfunction; CRSwNP, chronic rhinosinusitis with nasal polyps; EVEREST, phase IV head-to-head trial of dupilumab versus omalizumab in CRSwNP; HBC, horizontal basal cell; ILC2, group 2 innate lymphoid cell; SC, sustentacular cell; STAT6, signal transducer and activator of transcription 6; ST2, IL-33 receptor; TDI, threshold–discrimination–identification composite score; TSLP, thymic stromal lymphopoietin; UPSIT, University of Pennsylvania Smell Identification Test.
